# User Evaluation of a Chat-Based Instant Messaging Support Health Education Program for Patients With Chronic Kidney Disease: Preliminary Findings of a Formative Study

**DOI:** 10.2196/45484

**Published:** 2023-09-19

**Authors:** Nai-Jung Chen, Chiu-Mieh Huang, Ching-Chih Fan, Li-Ting Lu, Fen-He Lin, Jung-Yu Liao, Jong-Long Guo

**Affiliations:** 1 Department of Health Promotion and Health Education College of Education National Taiwan Normal University Taipei Taiwan; 2 Department of Nursing Taiwan Adventist Hospital Taipei Taiwan; 3 Institute of Clinical Nursing College of Nursing National Yang Ming Chiao Tung University Taipei Taiwan; 4 Department of Community Medicine En Chu Kong Hospital New Taipei Taiwan; 5 Department of Nursing University of Kang Ning Taipei Taiwan

**Keywords:** chronic kidney disease, chatbot, health education, push notification, users’ evaluation

## Abstract

**Background:**

Artificial intelligence–driven chatbots are increasingly being used in health care, but few chat-based instant messaging support health education programs are designed for patients with chronic kidney disease (CKD) to evaluate their effectiveness. In addition, limited research exists on the usage of chat-based programs among patients with CKD, particularly those that integrate a chatbot aimed at enhancing the communication ability and disease-specific knowledge of patients.

**Objective:**

The objective of this formative study is to gather the data necessary to develop an intervention program of chat-based instant messaging support health education for patients with CKD. Participants’ user experiences will form the basis for program design improvements.

**Methods:**

Data were collected from April to November 2020 using a structured questionnaire. A pre-post design was used, and a total of 60 patients consented to join the 3-month program. Among them, 55 successfully completed the study measurements. The System Usability Scale was used for participant evaluations of the usability of the chat-based program.

**Results:**

Paired *t* tests revealed significant differences before and after intervention for communicative literacy (*t*_54_=3.99; *P<*.001) and CKD-specific disease knowledge (*t*_54_=7.54; *P*<.001). Within disease knowledge, significant differences were observed in the aspects of CKD basic knowledge (**t*_5_*_4_=3.46; *P*=.001), lifestyle (*t*_54_=3.83; *P*=.001), dietary intake (*t*_54_=5.51; *P*<.001), and medication (*t*_54_=4.17; *P*=.001). However, no significant difference was found in the aspect of disease prevention. Subgroup analysis revealed that while the findings among male participants were similar to those of the main sample, this was not the case among female participants.

**Conclusions:**

The findings reveal that a chat-based instant messaging support health education program may be effective for middle-aged and older patients with CKD. The use of a chat-based program with multiple promoting approaches is promising, and users’ evaluation is satisfactory.

**Trial Registration:**

ClinicalTrials.gov NCT05665517; https://clinicaltrials.gov/study/NCT05665517

## Introduction

### Background

In 2017, 697.5 million people were reported to have chronic kidney disease (CKD), suggesting a global prevalence rate of 9.1% [1]. CKD’s prevalence is even higher in some developed countries. For example, more than 1 in 7 adults, that is 37 million or 15%, in the United States were estimated to have CKD. Unfortunately, as many as 9 of 10 adults with CKD are unaware [2]. When patients have CKD, their kidneys will gradually damage, which potentially leads to high blood pressure, heart disease, stroke, and early death. A previous cohort study found that the prevalence rate of CKD in Taiwan was 11.9%, with the total number of CKD patients estimated to exceed 2 million. However, only 3.5% (n=16,180) of patients were aware that they have CKD, highlighting the significance and urgency of health education for patients with CKD in Taiwan to help them manage their prognosis [3]. Health literacy has been defined by the World Health Organization as the cognitive and social skills that determine the motivation and ability of individuals to gain access to, understand, and use information in ways that promote and maintain good health [4]. Health literacy is critical not only for patients’ health care, disease prevention, and health promotion but also to maintain or improve their quality of life [5,6]. Studies have also indicated that health literacy is a determinant of patients’ disease management [7,8].

After a proper understanding and identification of health literacy among patients with CKD, an early intervention to improve health literacy and thereby delay CKD’s progression into end-stage renal disease is recommended. Thus, patients with CKD require further health literacy intervention studies to improve the prognosis of their disease. Despite the increased awareness of the importance of health literacy–based education programs for patients with CKD, few effective studies targeting patients with CKD have been conducted. A previous review to identify promising intervention targets and strategies in patients with CKD found that heterogeneity, low quality, and focus on kidney failure largely impede the identification of promising intervention targets and strategies for patients with low health literacy. The review suggested that more high-quality studies on early CKD stages are needed to identify targets and strategies to prevent disease deterioration among patients with CKD in the future [9].

Health literacy interventions are especially critical for diseases that require a high level of patients involvement to improve their disease management, such as CKD. CKD management is complex and requires patients to comprehend numerous issues including blood pressure, blood sugar, weight, cholesterol, diet, exercise, dialysis, medication, and both adherence and responsive actions. Patients with CKD often navigate the health care facilities and interact with different health care providers. Health literacy interventions aimed at improving patients’ self-management knowledge and ability could be incredibly useful in patients with CKD at both the early and end stages. A Cochrane systematic review protocol suggested that health literacy interventions aim to mitigate the effects of low health literacy; facilitate literacy skill building; and improve knowledge about disease and treatment, self-care, and comprehension skills [10].

Following the advancements in information and communication technology, its application to improve patients’ health outcomes has become increasingly popular. The WHO has stated that digital health, specifically mobile health, has improved the quality and coverage of care; increased access to health information, services, and skills; and promoted positive changes in health behaviors to prevent the onset of acute and chronic diseases [11]. For example, a mobile health study on 932 patients with CKD revealed that about 70% used the internet, email, or smartphones, while 35% used apps to understand disease conditions, find nutrition or diet information, or access their medical records [12]. This indicates that the use of information and communication technology in health literacy interventions for patients with CKD is possible and feasible. Recently, the use of chatbots in health care and education studies has attracted widespread attention. A chatbot is a software program that interacts with users through natural language dialogue and is widely used in instant messaging applications [13]. It provides health information for patients, and its usage is estimated to increase by more than 30% by 2024. Chatbots have instant messaging app functions and are easy to use [14,15].

Chatbot-based intervention studies on patients with CKD are scarce, highlighting the need for further research. Communicative literacy is one aspect of health literacy [16]. In addition to knowledge about the disease, patients’ ability to interpret health care professionals’ messages can contribute to better disease management and control [7]. In this study, we focus on communicative literacy, which refers to CKD-specific health literacy, representing patients’ ability to understand advice and information provided by physicians or nurses. We did not include functional literacy because, as the participants simultaneously received regular patient education from the hospital’s nephrology staff, the functional literacy intervention effect may have been confounded. Critical literacy refers to advanced cognitive skills used to critically analyze information and acquire control over life events and situations. However, as a chat-based instant messaging system provided health information in response to patients’ queries and patients were not expected to engage in critical analysis of the information provided, critical literacy was also excluded.

A previous study indicated that CKD-specific disease knowledge includes various aspects of CKD basic knowledge, disease prevention, lifestyle, dietary intake, and medication [17]. The significance of CKD basic knowledge has been consistently recognized in previous research focused on patients with CKD [18,19]. Prior studies have revealed that lifestyle modification can prevent the progression of CKD [20]. Lifestyle modifications, including changes in dietary intake, physical activity, and substance use, can help prevent the progression of CKD [21]. Low protein diets have been proposed for participants with CKD in order to slow the illness progression, thereby delaying the onset of renal replacement therapy [22]. Poor medication adherence among patients with CKD has been identified as a crucial risk factor for the progression of the disease [23].

### Study Purpose

The purpose of this formative study is to gather the data required to develop an intervention program of chat-based instant messaging support health education (CIM-SHE) for patients with CKD. The user experiences of participants were gathered as a basis for program design improvement.

## Methods

### Participants and Recruitment

A pre-post intervention design was used in this study. Participants were recruited from the nephrology outpatient department at a regional teaching hospital in Northern Taiwan. The selection criteria were as follows: participants had to (1) be patients with CKD aged 20 years or older, (2) be able to communicate in Mandarin or Taiwanese, (3) own a smartphone with an instant messaging application installed, and (4) be willing to participant in a 3-month intervention. Participants who had communication barriers (either physically or cognitively) or were participating in other intervention programs or services were excluded from this study.

**Figure 1:**
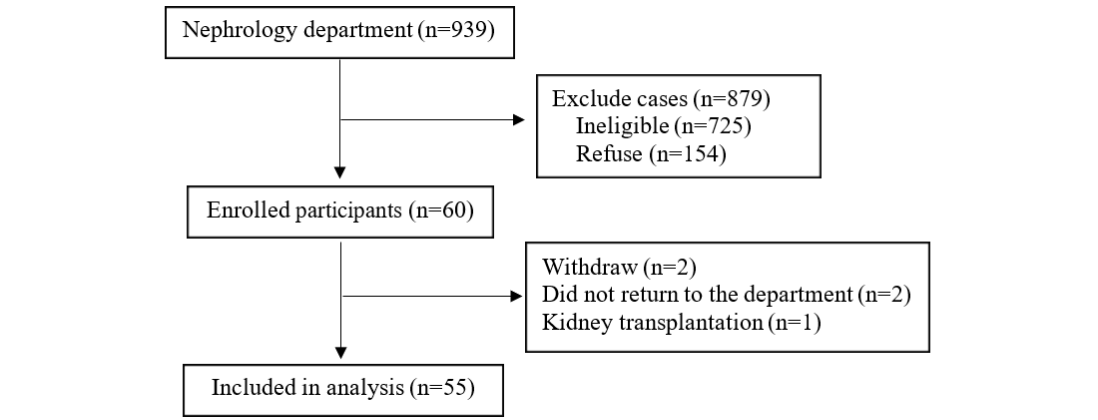
Study enrollment flowchart.

The participants were selected patients who met the sampling criteria. The recruitment period was from April to November 2020. Recruitment took place at a nephrology outpatient department. During the recruitment period, a total of 939 patients, not all of whom were patients with CKD, sought medical care. We initially planned to recruit 60 patients to test the CIM-SHE and gather their user experiences for program improvement. Despite the prevailing circumstances of the pandemic and administrative burdens, we were able to obtain consent from 60 patients to participate in the 3-month study over an 8-month period (from April to November 2020). Among the 60 participants, 2 withdrew from the study, 2 did not return to the department, and 1 received a kidney transplant. Thus, 55 patients completed repeated measurements after using the CIM-SHE for 3 months. The study enrollment flowchart is illustrated in Figure 1.

### Program Development and Implementation

A CIM-SHE program was developed to improve participants’ health literacy related to communication ability and disease-specific knowledge.

### CIM-SHE Program

The chatbot in this study was designed using the Line platform to provide a chat-based instant messaging service. To develop the content of the CIM-SHE program, we interviewed 10 patients with CKD and compiled their questions regarding disease management. Additionally, the *Handbook of CKD Health Management* [24] and *the CKD Clinical Diagnosis and Treatment Guidelines* [25] were used to develop 350 question and answer (Q&A) corpora that were reviewed by a senior nephrology attending physician to justify their appropriateness and correctness.

**Figure 2:**
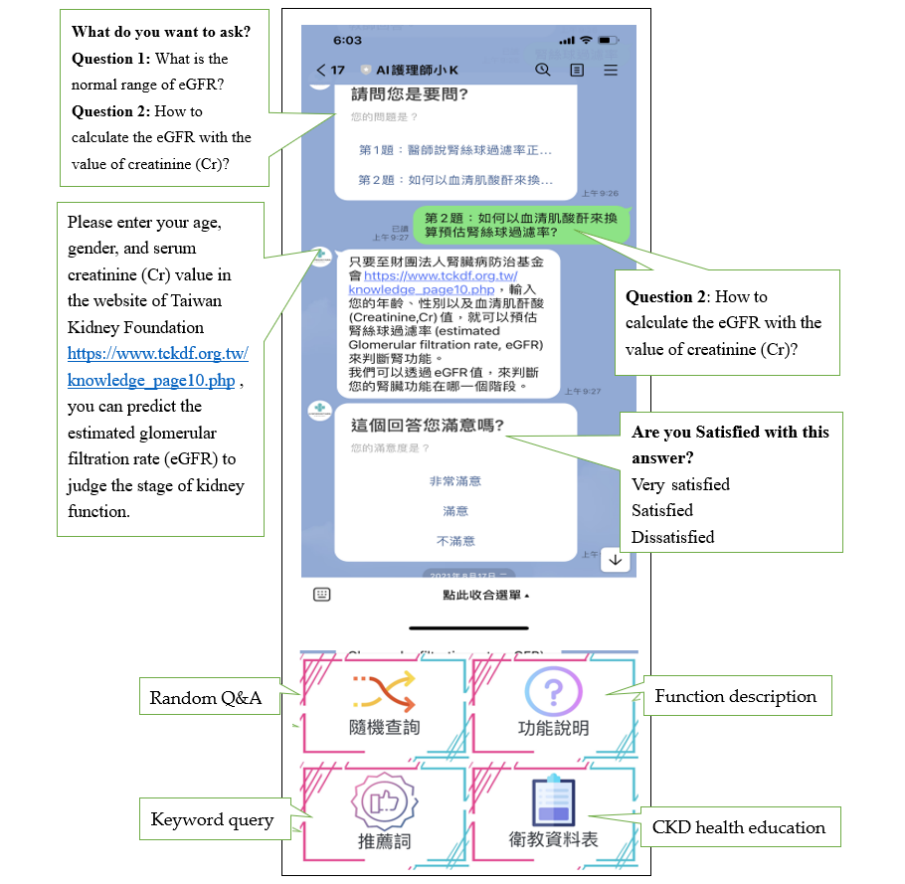
Interface of the chat-based instant messaging support health education program. CKD: chronic kidney disease; eGFR: estimated glomerular filtration rate; Q&A: question and answer.

The CIM-SHE contains four interfaces: (1) random Q&A, (2) function description, (3) keyword query, and (4) CKD health education (Figure 2).

### Promoting Approaches Adjunct to CIM-SHE

We used 4 promoting approaches to maximize participants’ engagement. First, participants could use the keyword query to ask questions related to CKD health information by inputting a keyword. For example, “a high-phosphorus diet” could be entered to search for information about high-phosphorus foods. The chatbot system would then present several possible questions based on fuzzy comparison from which the participants could choose the most pertinent one. Then, the corresponding answer would be displayed. If there were no matched questions or answers corresponding to the keyword query in the 350 Q&A database, the following message would appear: “Please wait; we will reply as soon as possible.” A head nurse of a nephrology ward responds to these queries personally. As the manager of this chatbot, the nurse immediately receives notifications about queries requiring responses.

The second approach is the “Line push notification.” The chatbot manager collects health information associated with CKD care and broadcasts it on the Line app (LINE Corporation). This approach prompts users with contextually tailored messages to increase their engagement [26]. The third approach is awarding participants to enhance their engagement. Three offered awards are for asking problem-discovery questions, the most frequently asked questions, and the best usage of the CIM-SHE system in order to encourage participants’ further involvement. The problem-discovery award is awarded to the participant who asks novel questions associated with their daily life. These questions may provoke creative and insightful discussions to solve problems. The award for most frequently asked questions is given to the participant who asks the most questions. The participant who most often interacts with the chatbot system and promptly responds to Line push notifications is awarded the best usage award.

The chatbot manager also actively interacts with participants during the intervention to improve their learning motivation. In addition, participants received a gift certificate of NT $300 (approximately US $10) after completing the pretest, intervention, and posttest. The fourth approach is to allow participants to express their level of satisfaction. Whenever a participant asks a question, after the chatbot answers, it would be followed by an evaluation question: “Are you satisfied with this answer?” Three options included “very satisfied,” “satisfied,” and “dissatisfied.” If the participant responds “dissatisfied,” the manager would promptly improve the answer or clarify the context of the participants’ questions and provide modified responses to improve the intervention’s effectiveness.

### Measurements

The demographic variables include age, gender, marital status, education level, stage of CKD, number of comorbidities, and BMI. Assessment tools of CKD-specific communicative literacy and knowledge were applied to measure the user experiences after receiving permission, and the System Usability Scale (SUS) was used to evaluate the acceptability of the participants using the CIM-SHE [17].

### CKD-Specific Communicative Literacy and Knowledge Assessment

In this study, communicative literacy refers to the communication skills used to assess adherence to physicians’ and nurses’ CKD management advice. This tool comprises two parts: (1) CKD-specific health literacy (communicative literacy) and (2) CKD-specific disease knowledge. A sample scenario is the doctor says: “When a patient with CKD has severe edema, it is necessary to limit the water intake. The daily water intake is the urine volume of the previous day plus 500-700 c.c.” After participants read this scenario, the following challenge question is presented: “If your urine volume was 700 c.c. the previous day, which option is more appropriate for your water intake today?”

The original CKD-specific disease knowledge scale included 13 items [27]; we added 2 items that reflect the specific eating habits of our participants, as revealed by the interview process, which resulted in 15 total items including 3 on CKD basic knowledge, 2 on disease prevention, 4 on lifestyle, 4 on dietary intake, and 2 on medication. Participants scored 1 point for each correct answer and 0 points for wrong answers.

These measurements have previously been applied to patients with CKD [27], with the Kuder–Richardson Formula 20 values of reliability for health literacy and disease-specific knowledge being 0.78 and 0.76, respectively. In this study, the Kuder–Richardson Formula 20 values of reliability for communicative literacy and CKD-specific disease knowledge were 0.71 and 0.65, respectively.

### SUS

SUS was developed to subjectively evaluate the user's operating system to understand and ensure its usability and quality [28]. The scale consisted of 10 items rated on a Likert-type scale ranging from 1 (strongly disagree) to 5 (strongly agree). In this study, items 1-5 and 6-10 were considered positive and negative, respectively. For positive items, we subtracted 1 point and for negative items, we subtracted 5 points from the score of the items. After the scores for each item were calculated, we added all the scores and multiplied by 2.5 to acquire the sum score. Sauro [29] indicated that if the average score of SUS is 68, it can be used to justify the product qualification. The Cronbach α coefficient was .91.

### Data Collection and Analyses

Owing to the COVID-19 pandemic during the data collection period, the CKD-specific assessment tool and SUS were conducted either face-to-face or through telephonic interviews, depending on the participants’ willingness. The participants were referred by a nephrologist to our research team in the outpatient department. The time points of measurement were based on the patient’s routine visits. A patient with CKD would have a follow-up visit every 3 months. At the baseline, the participants completed the pretest and were invited to participate in the study. The research team met face-to-face with the participants to advise them on operating the chatbot and interacting with the instructor and other participants. After 1 week of using the chatbot, the first SUS measurement was administered; the second and third SUS measurements were administered in the 4th and 12th week of intervention. After the intervention was completed, the posttest was administered.

Data analysis was performed using SPSS (version 23.0; IBM Corp). Paired *t* tests were used to compare the pre and post changes in participants’ communicative literacy and CKD-specific disease knowledge. Differences between male and female participants as indicators of effectiveness were also examined. The generalized estimating equation was used to evaluate the changes in SUS scores at 3 time points.

### Ethical Considerations

This research was approved by the ethics committee of a regional teaching hospital in Taipei, Taiwan (approval 109-E-04). Participants received information about various aspects of the study, explained to them their rights on voluntary participation, and withdrawal from the study at any time as well as their privacy and confidentiality rights. Participants provided prior written informed consent.

## Results

### Background Information

As shown in Table 1, the mean age of the participants was 54.69 (SD 10.35) years, and 64% (35/55) were male participants. The top 3 comorbidities were hypertension (n=43, 78%), hyperlipidemia (n=26, 47%), and diabetes (n=25, 45%), with 17 (31%) and 24 (44%) participants being overweight and obese, respectively.

**Table 1:** Background information (N=55).

Variables		Values
Age (years), mean (SD)		54.69 (10.35)
**Gender, n (%)**		
	Male	35 (64)
	Female	20 (36)
**Marital status, n (%)**		
	Married	17 (31)
	Other	38 (69)
**Educational level,** **n (%)**		
	High school or below	20 (36)
	College or higher	35 (64)
**Number of comorbidities, n (%)**		
	1	6 (11)
	2	22 (40)
	≥3	27 (49)
**Top 5 comorbidities (multiple choice), n (%)**		
	Hypertension	43 (78)
	Hyperlipidemia	26 (47)
	Diabetes	25 (45)
	Gout	19 (34)
	Coronary heart disease	8 (14)
**BMI (kg/m^2^), n (%)**		
	Underweight (BMI<24)	14 (25)
	Overweight (BMI 24-27)	17 (31)
	Mild obesity (BMI 27-30)	9 (16)
	Moderate obesity (BMI 30-35)	8 (14)
	Severe obesity (BMI≥35)	7 (12)

### Improvements in Communicative Literacy and CKD-Specific Disease Knowledge

The paired *t* tests revealed the significant differences before and after intervention on communicative literacy (*t*_54_=3.99; *P*<.001) and CKD-specific disease knowledge (*t*_54_=7.54; *P*<.001). Within CKD-specific disease knowledge, significant differences were observed in the aspects of CKD basic knowledge (*t*_54_=3.46; *P*=.001), lifestyle (*t*_54_=3.83; *P*=.001), dietary intake (*t*_54_=5.51; *P*<.001), and medication (*t*_54_=4.17; *P*=.001), as shown in Table 2. However, no significant difference was found in the aspect of disease prevention. Subgroup analysis revealed that male participants showed similar findings to the main analysis. However, female participants did not exhibit significant differences in 3 aspects of CKD-specific disease knowledge, including disease prevention (*t*_54_=1.00; *P*>.005), lifestyle (*t*_54_=1.42; *P*>.005), and medication (*t*_54_=1.75; *P*>.005), between pretest and posttest measurements.

**Table 2:** Changes before and after intervention on communicative literacy and CKD-specific disease knowledge for all, male, and female patients with CKD.

Variable			All (N=55)									Male (n=35)								Female (n=20)						
			Mean (SD)				*t* test (*df*)		*P* value		Mean (SD)					*t* test (*df*)		*P* value	Mean (SD)					*t* test (*df*)		*P* value
			Pretest		Posttest						Pretest			Posttest					Pretest			Posttest				
**Health literacy**																										
	Communicative literacy	3.40 (0.83)		3.87 (0.34)		3.99 (54)		<.001		3.46 (0.82)			3.86 (0.36)		2.79 (54)		.01		3.30 (0.87)		3.90 (0.31)		2.85 (54)		.01	
**CKD^a^-specific disease knowledge**			12.93 (1.71)		14.60 (0.66)		7.54 (54)		<.001		13.11 (1.21)			14.71 (0.52)		8.10 (54)		<.001	12.60 (2.35)			14.40 (0.82)		3.52 (54)		.002
	CKD basic knowledge	2.69 (0.57)		2.98 (0.14)		3.46		.001		2.80 (0.41)			3.00 (0.00)		2.92 (54)		.006		2.50 (0.76)		2.95 (0.22)		2.65 (54)		.01	
	Prevention	1.93 (0.33)		2.00 (0.00)		1.66 (54)		.10		1.94 (0.24)			2.00 (0.00)		1.44 (54)		.16		1.90 (0.45)		2.00 (0.00)		1.00 (54)		.33	
	Lifestyle	3.51 (0.72)		3.87 (0.34)		3.83 (54)		<.001		3.54 (0.70)			3.97 (0.17)		3.87 (54)		<.001		3.45 (0.76)		3.70 (0.47)		1.42 (54)		.17	
	Dietary intake	3.27 (0.80)		3.87 (0.34)		5.51 (54)		<.001		3.34 (0.77)			3.86 (0.36)		3.90 (54)		<.001		3.15 (0.88)		3.90 (0.31)		3.94 (54)		.001	
	Medication	1.53 (0.54)		1.87 (0.34)		4.17 (54)		<.001		1.49 (0.56)			1.89 (0.32)		3.92 (54)		<.001		1.60 (0.50)		1.85 (0.37)		1.75 (54)		.01	

^a^CKD: chronic kidney disease.

### Improvements in SUS

The SUS scores were calculated at 3 time points that resulted in the time 1 (mean score 71.19, SD 15.31), time 2 (mean score 76.42, SD 16.29), and time 3 (mean score 80.23, SD 13.39) measurements, respectively. These 3 measurements progressively increased over time, indicating that the CIM-SHE in this study was well used among participants.

**Figure 3:**
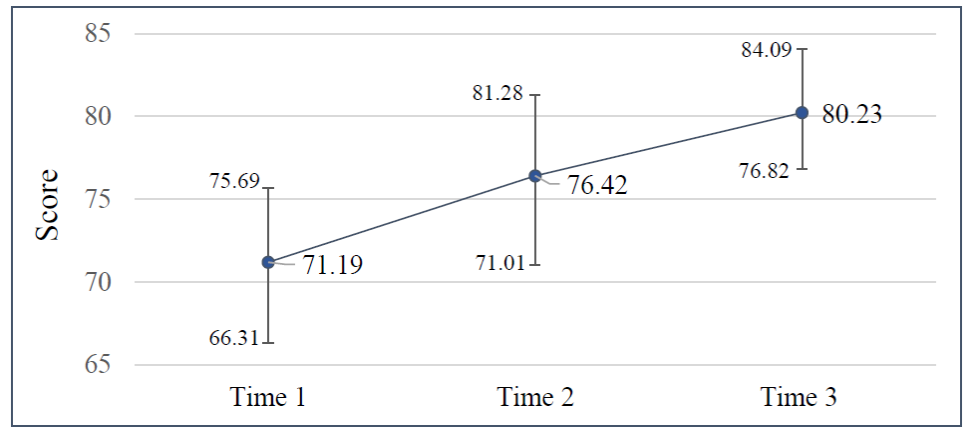
Mean System Usability Scale scores at 3 time points.

The SUS measurement results revealed that the scores in the second (coefficient=0.23; Wald *χ*_1_^2^=10.3; *P*=.001) and third (coefficient=0.38; Wald *χ*_1_^2^=22.3; *P*<.001) measurements showed significant improvement compared to the first measurement scores (Figure 3).

## Discussion

### Principal Findings

Our study revealed that a chat-based instant messaging support program using 4 approaches to enhance communicative literacy and CKD-specific disease knowledge in patients may hold promise. The system usability of the CIM-SHE was measured using the SUS score, and the participants’ evaluations indicated a high level of system usability. The mean SUS score was 71.19 (SD 15.31) in the first week of use, increased to 76.42 (SD 16.29) after 1 month of use, and further increased to 80.23 (SD 13.39) after 3 months of use. According to Bangor et al [30], a SUS score of 50 indicates “OK,” a score below 52 indicates that the system needs improvement, a score exceeding 73 indicates the system is “good,” and a score above 86 is considered “excellent.” A previous study recruited 22 patients with CKD to assess the usability of an app (Dialysis Guide; The Ottawa Hospital Research Institute) by administering the SUS. The results revealed that the mean SUS score was 66.82 (SD 14.54) [31]. The SUS provides a global view of subjective assessment of usability and is often used in comparisons of usability between systems. Thus, it is likely that the CIM-SHE will generally be accepted by patients with CKD.

We observed significant improvements in communicative literacy and CKD-specific disease knowledge after 2 months of usage, with the exception of the lifestyle aspect. The observed effects were similar to that of previous mobile phone–based interventions for disease knowledge of patients with CKD [32]. We ensured the engagement of participants through 4 promotional approaches. The first approach involved seizing the opportunity to teach participants how to use appropriate words to ask questions after they downloaded and installed the chatbot app. The second approach involved our research team providing push notifications regarding multiple disease management messages each week so that participants could receive timely updates and information. Previous studies have indicated that push notifications are an effective information dissemination strategy [33]. A key requirement of apps is that they should keep their mobile users up-to-date as changes occur in relation to some issues of interest that help users engage in using these apps. Our second approach appears to have achieved the effect. The third approach was also successful because our instructor found that rewards have an influence on participants’ motivation for engaging in learning, which is consistent with the findings of a previous literature review [34]. The fourth approach involved allowing participants to evaluate the quality of answers to the questions they asked; it also allowed dissatisfied participants to privately use the Line service to receive more individualized answers. During the intervention process, we also felt that the abovementioned 4 approaches could effectively increase usage among participants through the frequent use of the chatbot, which contributed to the intervention’s effectiveness. Individualized instruction for participants of different abilities within the same program is crucial, which is a finding that is supported by previous literature [35].

Combining a chatbot with push notifications and feedback rewards can significantly improve communicative literacy and CKD-specific disease knowledge. Our findings are similar to those of a previous study [36], which found that the chatbot can help participants understand several health issues and engage them in a good conversation. While the participants in the study were young adults, which differs from the middle-aged and elderly adults in this study, the effect and users' evaluation were both positive. The findings suggest that the developed chatbot is promising and may be used as an agent to increase communicative literacy and knowledge.

However, some participants informed the research team that the chatbot occasionally failed to provide appropriate answers, especially when dealing with complex questions. The chatbot performed relatively better when faced with straightforward inquiries owing to its specific path-following design and limited range of programmed scripts. If the user’s question is too complicated, the chatbot’s recognition and understanding abilities are challenged, leading to inaccurate responses. To address this issue, improvements in natural language processing comprehension and programming algorithms are necessary to enhance the chatbot’s question recognition capabilities [14].

A crucial feature of our chatbot is that in the process of creating the Q&As. The research team invested substantial effort into translating complex medical terms and narratives into simple vernacular language. When participants were asked about the meaning of the term “eGFR,” the chatbot provided the Chinese translation of “Estimated glomerular filtration rate” and the formula to assist them in comprehending the value calculation and its corresponding stage based on guidelines. Participants can input their values and the chatbot will provide corresponding recommendations. A structured and patient-specific teaching has been found to be superior to ad hoc or generalized teaching [37]. Our participants were given several choices and invited to select the aspects they preferred to read, such as CKD basic knowledge, disease prevention, lifestyle, dietary intake, and medication. They could arrange the learning sequence on their own or choose the content they were interested in. This design encourages participants to ask questions, read answers, and search for related health information.

Chatbots, by using their conversational feature, create scenarios that enhance user engagement and have been used in various aspects of disease management and health care, including cancer care [38], mental health [39], dementia [40], and fighting COVID-19 pandemic [41]. Given their successful use in various health care settings, chatbots have the potential to be integrated into clinical practice by working alongside health care providers to refine efficiencies and improve patient outcomes. In addition, health care providers should guide patients to find an appropriate chatbot to support their health needs. However, most chatbot studies have been conducted in the United States, and cultural context may influence the application of chatbots. As Taiwanese studies tend to focus on exploring the factors affecting technology acceptance of chatbots among older adults [42] or community consumers [43], further studies are needed to explore the cultural influence.

### Limitations

This study has the following limitations. First, we applied a single-group pretest-posttest design without including a control group, which limited the generalizability of the findings. However, it must be noted that the primary focus of this formative study is on user experience, rather than comparison. Second, the recruitment period was during the COVID-19 pandemic, resulting in a higher refusal rate (Figure 1) than during ordinary circumstances, which limited the sample size. The small sample size, subsequently, restricted the number of variables that could be included in statistical analysis. For instance, educational level was not included. Further studies are encouraged to increase sample size and incorporate factors influencing disease knowledge and health literacy into the statistical analysis.

### Conclusions

The system usability of the CIM-SHE was measured using the SUS score, and the study participants' evaluations indicated a high level of system usability. This study revealed promising results for a chat-based instant messaging support program that uses 4 promoting approaches to enhance communicative literacy and CKD-specific disease knowledge for middle-aged and older patients with CKD. Future studies could explore the effectiveness of using apps or other digital platforms as intervention tools to improve patients' communicative literacy and CKD-specific disease knowledge.

## Abbreviations

CIM-SHE: Chatbot-based instant messaging support health education
CKD: chronic kidney disease
Q&A: question and answer
SUS: System Usability Scale

## Data Availability

The data sets used and analyzed during this study are available from the corresponding authors on reasonable request.
